# BODY MASS INDEX AND OLDER WOMEN AND FEMMES EXPERIENCES WITH DISCRIMINATION IN THE HEALTHCARE SETTING

**DOI:** 10.1093/geroni/igae098.4134

**Published:** 2024-12-31

**Authors:** Tavia Roache, Lauren Brown, Amor Mathershed, Heaven Stewart, Leilani Benton, Mekiayla Singleton

**Affiliations:** University of Southern California, Los Angeles, California, United States; University of Southern California, Los Angeles, California, United States; University of Southern California, Los Angeles, California, United States; University of Southern California, Los Angeles, California, United States; University of Southern California, Los Angeles, California, United States; University of Southern California, Los Angeles, California, United States

## Abstract

BMI is used widely both within and outside of healthcare settings to understand health despite the contradictory evidence around the impact of obesity on mortality in middle age and older adulthood. We examine how BMI impacts reports of older white, Black and Latine women and femmes’ self-reports of ever experiencing discrimination in the health care setting over their lifetime using the Health and Retirement Study (HRS) leave behind questionnaire (n=7,614). We also examine if experiencing this type of discrimination in the healthcare setting impacts these women’s mental health. Having ever been unfairly denied health care or treatment was more commonly reported by older Black (n=1,059) and Latine (n=588) women/femmes relative to older white women. Regardless of race/ethnicity, women and femmes with a higher BMI have an increased likelihood of reporting being unfairly denied health care (OR=1.03, p=0.01). Women who report being unfairly denied treatment in the doctor’s office also report more depressive symptoms (ℬ=0.93; p< 0.001). Findings demonstrate that women with higher BMI are at increased risk of experiencing discrimination in a health care setting over their lifetime. This is especially true for Black and Latine women/femmes who are more likely to report discrimination. Anti-racist efforts in healthcare must also address unfair treatment of women and femmes based on their BMI.

